# Acetyl-CoA: An interplay between metabolism and epigenetics in cancer

**DOI:** 10.3389/fmmed.2022.1044585

**Published:** 2022-11-16

**Authors:** Yang Hao, Qin Yi, Xu XiaoWu, Chen WeiBo, Zu GuangChen, Chen XueMin

**Affiliations:** ^1^ Changzhou First People’s Hospital, The Third Affiliated Hospital of Suzhou University, Changzhou, China; ^2^ Department of Pancreatic and Hepatobiliary Surgery, Fudan University Shanghai Cancer Center, Shanghai, China; ^3^ Department of Oncology, Shanghai Medical College, Fudan University, Shanghai, China; ^4^ Pancreatic Cancer Institute, Fudan University, Shanghai, China

**Keywords:** acetyl-CoA, metabolism, epigenetics, cancer, histone acetylation

## Abstract

Due to its high mortality and severe economic burden, cancer has become one of the most difficult medical problems to solve today. As a key node in metabolism and the main producer of energy, acetyl-coenzyme A (acetyl-CoA) plays an important role in the invasion and migration of cancer. In this review, we discuss metabolic pathways involving acetyl-CoA, the targeted therapy of cancer through acetyl-CoA metabolic pathways and the roles of epigenetic modifications in cancer. In particular, we emphasize that the metabolic pathway of acetyl-CoA exerts a great impact in cancer; this process is very different from normal cells due to the “Warburg effect”. The concentration of acetyl-CoA is increased in the mitochondria of cancer cells to provide ATP for survival, hindering the growth of normal cells. Therefore, it may be possible to explore new feasible and more effective treatments through the acetyl-CoA metabolic pathway. In addition, a growing number of studies have shown that abnormal epigenetic modifications have been shown to play contributing roles in cancer formation and development. In most cancers, acetyl-CoA mediated acetylation promotes the growth of cancer cells. Thus, acetylation biomarkers can also be detected and serve as potential cancer prediction and prognostic markers.

## Introduction

With advancements in medical technology and the continuous deepening of research in recent decades, an increasing number of diagnostic and treatment methods have been applied for cancers to achieve better curative effects. Unfortunately, cancer remains one of the most fatal diseases. Among all human diseases, cancer poses the greatest clinical, social, and economic burdens because of cause-specific disability-adjusted life years. Additionally, and it is the second leading cause of death worldwide after ischemic heart diseases (Mattiuzzi and Lippi, 2019). Conventional treatments, including precise partial excision of tumors through surgery and targeted radiotherapy and chemotherapy have greatly reduced cancer mortality, but their results have not been entirely satisfactory. To this end, we are looking for cancer markers, many of which can mediate epigenetic modifications and promote the abnormal proliferation of cells and cancer development and maintenance (Rinaldi et al., 2018). Acetyl-CoA is a key metabolite whose metabolic pathway and the manner in which it controls epigenetic modification, may be leveraged to produce satisfactory therapeutic effects in cancer, which makes it worthy of further research and exploration.

## Acetyl-coenzyme A metabolic pathways

### Anabolism of acetyl-coenzyme A metabolism

**FIGURE 1:**
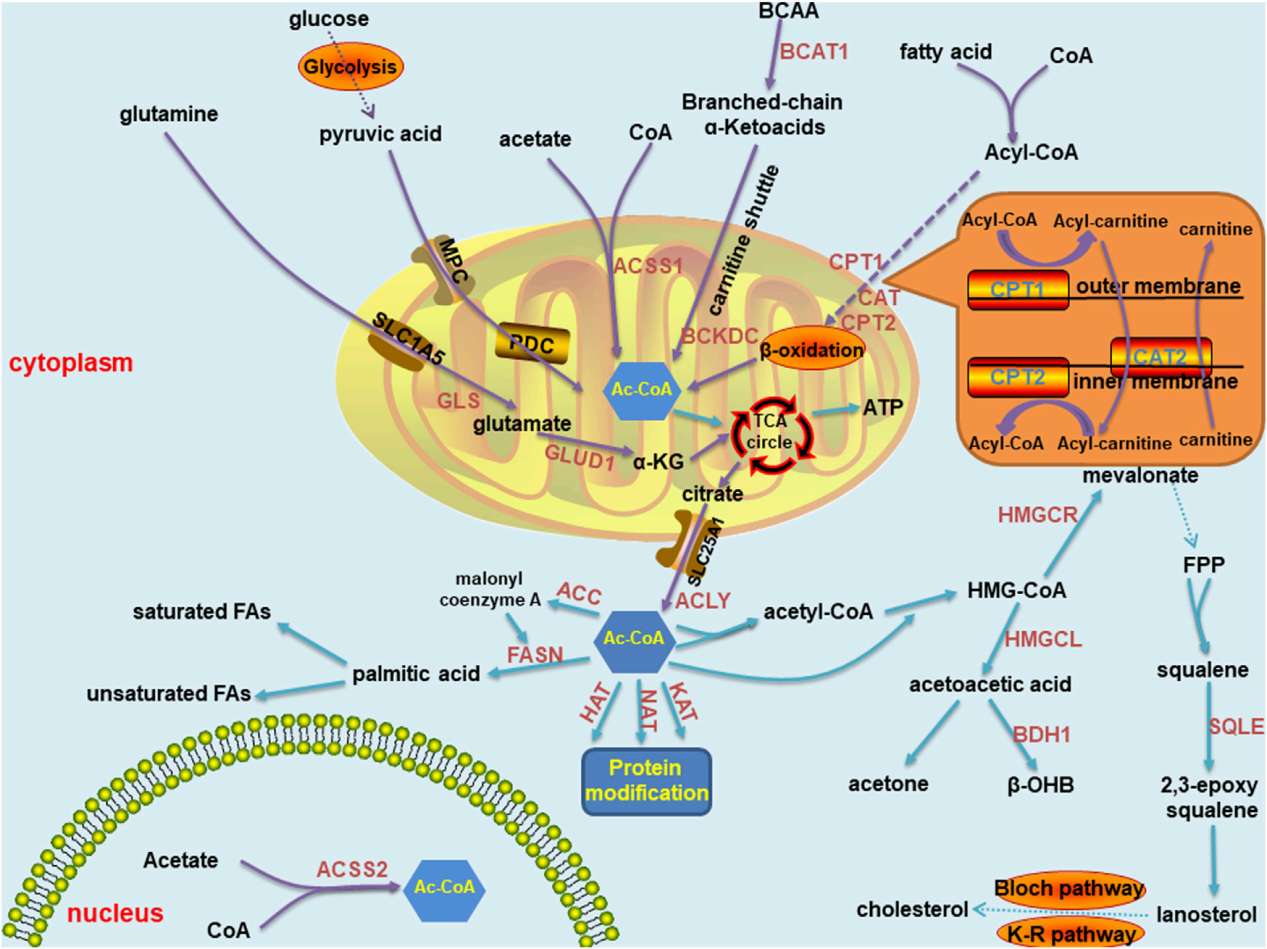
Summary of acetyl-CoA metabolic pathways in normal cells, including anabolism and catabolism. The synthetic pathways are marked with purple arrows, and the decomposition pathways are marked with blue arrows. Most of these pathways are located in mitochondria and cytoplasm, and a few in the nucleus. Key enzymes are marked in brown font. The carnitine shuttle route is shown separately in the orange box. Abbreviations: MPC, mitochondrial pyruvate carrier; PDC, pyruvate dehydrogenase complex;CPT, carnitine palmitoyltransferase; BCAT, branched-chain amino acid transaminase; BCKDC, branched-chain α-ketoacid dehydrogenase complex; ACSS, acyl-CoA short chain synthetase; GLS, glutaminase; GLUD, glutamate dehydrogenase; GPT, glutamate pyruvate aminotransferase; ACLY, ATP-citrate lyase; HMG-CoA, 3-hydroxy-3-methylglutaryl coenzyme A; HMGCS, 3-hydroxy-3-methylglutaryl coenzyme A synthase; HMGCL, 3-hydroxy-3-methylglutaryl coenzyme A lyase; HMGCR, 3-hydroxy-3-methylglutaryl coenzyme A reductase; BDH, β- hydroxybutyrate dehydrogenase; HAT, histone acetyltransferase; NAT, N-acetyltransferase; KAT, N-acetyltransferase; FPP: farnesyl pyrophosphate; SQLE, squalene cyclooxygenase; ACC, acetyl-CoA carboxylase; and FASN, fatty acid synthase.

Acetyl-CoA is the key metabolite in carbon metabolism. It is the most fundamental precursor in energy production, storage and utilization in all cells in the body. It also participates in a series of vital processes, such as lipid synthesis and protein acetylation, to maintain life activity. Therefore, acetyl-CoA is one of the most essential metabolic intermediates (Figure 1). Acetyl-CoA can be produced in various ways and is derived from a wide range of sources, mainly through carbohydrates, free fatty acids and branched-chain amino acids. First, in humans, ingested glucose is decomposed into pyruvate through glycolysis. Then, pyruvate is transported into mitochondria through a mitochondrial pyruvate carrier (MPC) and ultimately decarboxylated by the pyruvate dehydrogenase complex (PDC) to acetyl-CoA (Jones et al., 1993). Second, acyl coenzyme A (acyl-CoA) synthase catalyses free fatty acids and coenzyme A to produce acyl-CoA in the cytoplasm. Carnitine palmitoyltransferase-1 (CPT1) transfers acyl groups from acyl-CoA to L-carnitine in the outer membrane of mitochondria to synthesize acyl-carnitine esters. Acyl-carnitine esters enter mitochondria through an exchange with free L-carnitine by carnitine acyl-carnitine translocase (CAT) and are then transformed into acyl-CoA by carnitine palmitoyltransferase-2 (CPT2) in the inner mitochondrial membrane. Thus, acyl-CoA is processed through the β-oxidation pathway to form acetyl-CoA (Adeva-Andany et al., 2019). Third, branched chain amino acids (BCAAs) can be catalysed by branched-chain amino acid transaminase 1 (BCAT1) to form branched-chain α-ketoacid and α-ketoglutarate (Harris et al., 2005), which are transported into mitochondria through the carnitine shuttle (Violante et al., 2013). Therefore, α-ketoacid undergoes decarboxylation mediated by the branched-chain α-ketoacid dehydrogenase complex (BCKDC) to produce acetyl-CoA and other acyl-CoAs (Harris et al., 2005). Acetyl-CoA can also be synthesized from acetate and coenzyme A through catalysis mediated by acyl-CoA short chain synthases (ACSS) in mitochondria (ACSS1) and in both the cytoplasm as well as the nucleus (ACSS2) (Fujino et al., 2001; Wellen et al., 2009; Ariyannur et al., 2010). Moreover, glutamine is transferred into mitochondria by an SLC1A5 variant. There, it is converted to glutamate through glutaminase catalysis, and then converted to α-ketoglutarate by glutamate dehydrogenase 1 (GLUD1) or several mitochondrial aminotransferases, including glutamate pyruvate aminotransferase 2 (GPT2) and glutamate oxaloacetate aminotransferase 2. Then, α-ketoglutarate enters the tricarboxylic acid (TCA) cycle, where it undergoes reductive carboxylation to generate citrate and is transported to the cytoplasm by SLC25A1 (Yoo et al., 2020). As a result, acetyl-CoA is generated from citrate by ATP-citrate lyase (ACLY) (Zaidi et al., 2012).

### Catabolism of acetyl-coenzyme A

Similar to the prolific synthesis pathways of acetyl-CoA, its catabolic pathways are diverse. The most common pathway of the acetyl-CoA catabolic pathway in circulation is ATP production through the mitochondrial TCA cycle (Akram, 2014). Moreover, certain cells, including liver cells, can synthesize ketones through acetyl-CoA to meet energy metabolism requirements. Mitochondrial hydroxyl-methylglutaryl coenzyme A synthase (HMGCS) condenses acetyl-CoA with acetoacetyl-CoA to form hydroxyl-methylglutaryl coenzyme A (HMG-CoA), through which hydroxyl-methylglutaryl coenzyme A lyase (HMGCL) releases acetoacetic acid. Acetoacetic acid is the precursor of two other common circulating ketone bodies, acetone and β-hydroxybutyric acid. Most acetoacetic acid is further metabolized to β-hydroxybutyric acid through β-hydroxybutyrate dehydrogenase (BDH) (Newman and Verdin, 2014). Acetyl-CoA in the cytoplasm can be used to conduct protein modification by histone acetyltransferases (HATs), N-acetyltransferases (NATs) and lysine acetyltransferases (KATs) (Drazic et al., 2016). In the cytoplasm of liver cells, acetyl-CoA can also be hydrolyzed by acyl-CoA thioesterase 12 (ACOT12) to coenzyme A and acetate (Suematsu et al., 2001; Kirkby et al., 2010). Similar to the early steps in ketone synthesis, two acetyl-CoA molecules in the cytoplasm condense to form acetyl-CoA, which reacts with a third acetyl-CoA to form HMG-CoA. HMG-CoA is reduced to mevalonate by hydroxyl-methylglutaryl coenzyme A reductase (HMGCR). A series of enzyme reactions convert mevalonate into farnesyl pyrophosphate. The condensation reaction of two farnesyl pyrophosphate molecules generates squalene. Squalene is oxidized into 2, 3-epoxy squalene by squalene cyclo-oxygenase (SQLE). Cyclo-oxygenated squalene is cyclized to lanosterol, and enters the Bloch pathway, the Kandutsch-Russell pathway or a mixed pathway to produce cholesterol (Huang et al., 2020). Acetyl-CoA can also be converted to malonyl coenzyme A by acetyl-CoA carboxylase and then coupled with acetyl-CoA to an acyl carrier protein domain in the multifunctional enzyme fatty acid synthase (FASN). Acetyl groups are repeatedly condensed to form the basic 16-carbon saturated fatty acid: palmitic acid. In mammalian cells, palmitic acid can be elongated and desaturated to produce various saturated and unsaturated fatty acids (Baenke et al., 2013).

### Abnormal regulation of acetyl-coenzyme A metabolism

**FIGURE 2:**
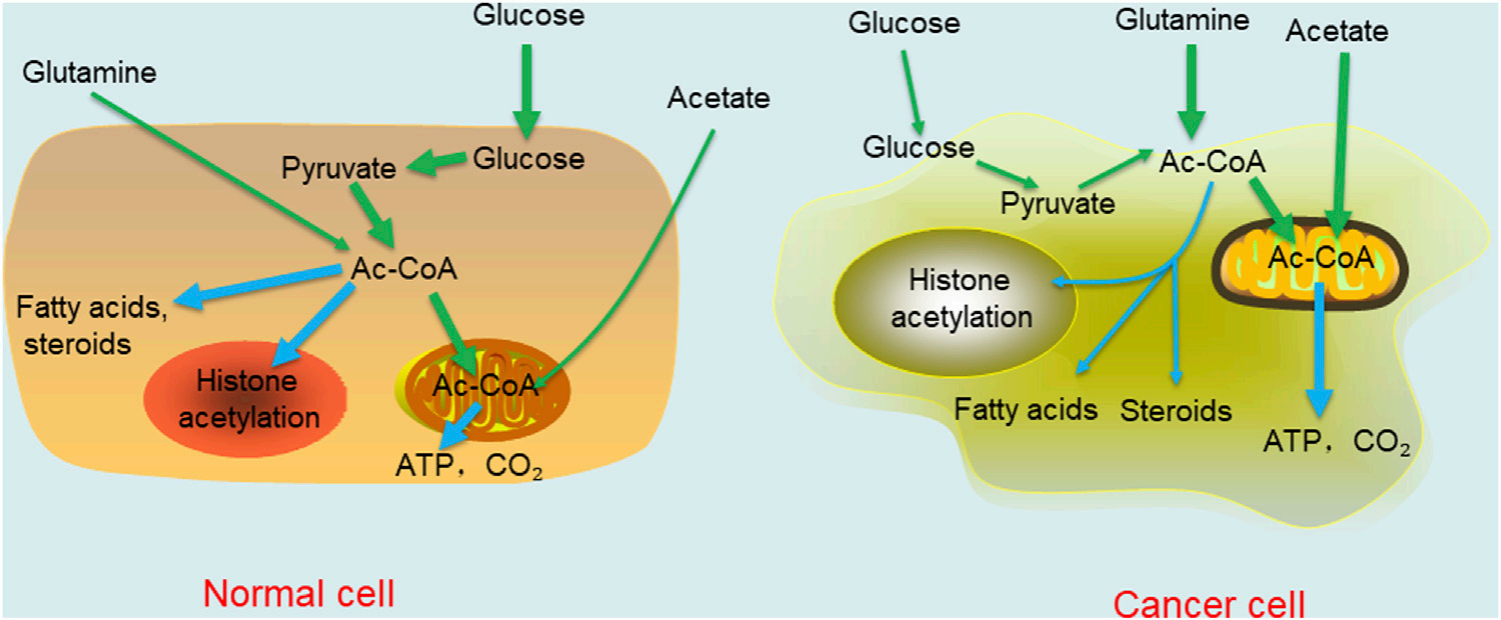
Comparison of acetyl-CoA metabolism in normal differentiated cell and cancer cell. The thickness of the line represents the degree of the reaction.

Changes in the external environment of the body affect the microenvironment of the body. Therefore, as a key node of metabolism in the body, acetyl-CoA changes in response to microenvironmental changes. In this section, we focus on the metabolic regulation of acetyl-CoA in cancers. Under aerobic conditions, noncancer cells mainly oxidize most of the pyruvate produced by glycolysis to carbon dioxide through the TCA cycle and synthesize a small portion of it into lactic acid in the cytoplasm. In contrast to normal tissue, the Warburg effect in cancer cells leads to the production of large amounts of lactic acid, resulting in glucose deprivation even when there is sufficient oxygen (Liberti and Locasale, 2016). When the human body is in a normal state, most acetyl-CoA concentrates in the cytoplasm, is catalysed into fatty acids and steroids, or is directed into the nucleus to promote histone acetylation. In cancer cells, the situation is reversed. With or without oxygen, cancer cells tend to ferment glucose into lactic acid in a process called “aerobic glycolysis”. As a result, there is a relative shortage of carbohydrates or glucose in normal tissues and cells, which must change from growth to survival mode. Specifically, high acetyl-CoA levels in cells are concentrated in mitochondria because this metabolite is needed for the oxidation reactions required to synthesize ATP to be used as cellular energy. In liver cells, mitochondrial acetyl-CoA is more commonly consumed in ketone synthesis, and then used as an alternative fuel source through β-oxidation in brain and heart cells (McGarry a and Foster, 1980). Under this condition, a low nuclear cytoplasmic acetyl-CoA level limits fatty acid synthesis, histone acetylation, and other important growth-related processes to the detriment of cell metabolism and growth. Moreover, the supply of oxygen greatly affects the metabolism of acetyl-CoA. Under normal conditions, that is, when oxygen is abundant, acetyl-CoA is derived mainly from glucose oxidation. In cancer, in which hypoxic conditions are caused by the compression of proliferating cancer cells, acetate and glutamine are the main sources of acetyl-CoA (Metallo et al., 2012; Kamphorst et al., 2014) (Figure 2). In addition, due to an increased fermentation rate and poor perfusion in cancer, the levels of lactic acid increase and cannot be effectively diffused in the tumor microenvironment. This results in a decrease in the pH of the tumor microenvironment (Estrella et al., 2013). Interestingly, despite acidic extracellular micro-environments arising as a result of high local lactate concentrations, the actual intracellular pH is actual somewhat alkaline relative to that of normal cells. Therefore, relatively high intracellular pH maintains the levels of acetylation, which is related to increased cell proliferation and cancer invasiveness, leading to adverse clinical outcomes (McBrian et al., 2013; Liu et al., 2020). Interestingly, a number of adverse microenvironments, such as those caused by glucose deprivation, hypoxia or low pH, do not disrupt the growth and proliferation of cancer cells. In contrast, cancer cells become more malignant and competitive than normal cells under these conditions. Moreover, *in vitro* experiments have revealed that when normal medium is changed to nutritional-depleted medium, the intracellular acetyl-CoA level in cultured human cancer cells is decreased before the ATP and NADH concentrations are decreased. In contrast, when cancer cells are cultured in enriched medium, various selective acetyl-CoA metabolic processes can effectively reduce the intracellular acetyl-CoA level (Mariño et al., 2014). These findings indicate that cancer cells may adapt to changes in the tumor microenvironment through self-regulation, which is the opposite response to that in normal cells. In general, cancer cells have evolved to overcome the harsh microenvironment, which further shows that there is a long way to go to cure cancers.

## Effects of acetyl-CoA metabolic pathways on cancer

Cancer has always been one of the most troublesome and insurmountable diseases in the medical field because of its high mortality. However, when cancer is found early, it can often be cured. Unfortunately, most malignant cancers show no obvious symptoms in the early stage. To date, four main modern medicinal treatments are used to treat cancers: surgery, chemotherapy, radiotherapy and immunotherapy. However, none of these methods is effective in advanced cancers. Therefore, finding a suitable and more effective treatment is the most urgent task for cancer researchers.

Scientists identified cancer as a metabolic disease 100 years after it was first described in modern medical terms. This discovery drove scholars to conduct metabolic research on cancers to identify the metabolic pathways that can be manipulated to inhibit cancer. However, cancer cells engage multiple active metabolic pathways and can quickly adapt to anti-metabolic chemotherapy drugs. Considering their adaptability, is there a way to suppress cancers? The answer to this question is “yes”. With the popularization of precision therapy in recent years, acetyl-CoA, as a key node in energy metabolism, has naturally become a hot spot in anticancer research.

### Cancer therapy mediated by the ATP-citrate lyase metabolic pathway

In addition to the Warburg effect, the most important metabolic feature of cancer is altered *de novo* lipid synthesis (Menendez and Lupu, 2007). The main raw material of lipid synthesis is cytoplasmic citrate, and the key enzyme is ACLY. Therefore, most studies on the influence of the acetyl-CoA metabolic pathway in cancer focus on ACLY metabolic pathways. ACLY is a homotetramer composed of four identical subunits with a molecular weight of approximately 120 kDa. Each peptide chain contains 1101 amino acid residues. It resides in the cytoplasm and nucleus and converts citrate and Co-A to acetyl-CoA and oxaloacetic acid (Elshourbagy et al., 1992). Previous research has shown that an increase in the acetyl-CoA synthesis rate promotes posttranslational histone acetylation at specific gene sites and accelerates cell division (Wellen et al., 2009; Shi and Tu, 2013). The effect of histone acetylation has been associated with the occurrence and progression of cancer, and many important discoveries have been made through experiments. For example, the production of ACLY-dependent acetyl-CoA was recently found to play an important role in the early stage of pancreatic cancer development, and once cancer is formed, ACSS2 is highly expressed. Even without ACLY activity, cancer cells can proliferate well. However, by targeting the acetyl-CoA-dependent pathway and using combined Bromodomain and extraterminal domain (BET) expression inhibition and statin therapy, scientists have shown that the proliferation of cancer cells and tumor growth can be inhibited (Carrer et al., 2019). A recent study showed that regulating ACLY activity to increase the production of acetyl-CoA can promote the occurrence and progression of breast cancer by maintaining histone H4 acetylation levels (Yang et al., 2020a). Another study showed that maintaining ACLY levels and regulating acetyl-CoA levels promote the proliferation, metastasis, and even drug resistance of cancer cells in nasopharyngeal cancer (Zheng et al., 2020). In addition, the ACLY levels in osteosarcoma, prostate cancer, cervical cancer and lung cancer cells were significantly higher than those in normal tissues. Through the upstream inhibition of miR-22, ACLY expression was downregulated, and more cells in cancer tissues underwent excellent differentiation (Xin et al., 2016). In acute myeloid leukemia, the overexpression of ACLY led to low cell differentiation and poor prognosis (Wang et al., 2019a).

**FIGURE 3:**
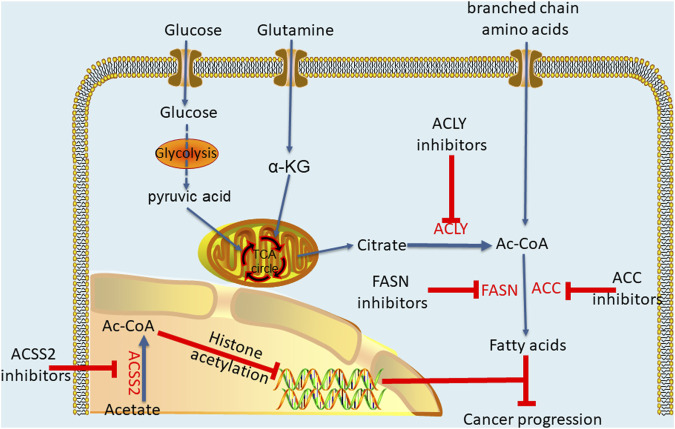
Key enzymes of acetyl-CoA metabolic pathway and effects of thier inhibitors.

ACLY plays a profound regulatory role in different cancers, and the dysregulation of ACLY expression is more likely to make cells cancerous. Interestingly, ACLY inhibitors have been used to treat metabolic disorders, and scientists are now trying to apply these inhibitors to cancer treatment (Granchi, 2018). Accordingly, through researches, many inhibitors are being developed as anticancer drugs, producing anticancer effects by inhibiting the metabolism pathwayof acetyl-CoA (Figure 3). The most well-known and the first discovered ACLY inhibitor is hydroxycitrate (HCA) (Sullivan et al., 1977). In both lung carcinoma and bladder cancer animal models, HCA and α-lipoic acid combination potentiated the efficacy of the tested anticancer drugs in reducing tumor growth and improving survival rate (Guais et al., 2012). Cucurbitacin B is another natural ACLY inhibitor found in cucumber, which inhibits the proliferation of prostate cancer cells by inducing apoptosis and does not have a significant impact on normal prostate epithelial cells (Gao et al., 2014). For synthetic ACLY inhibitors, there is still much room for development. As early as the end of the 20th century, SB-204990, a lactone prodrug of ACLY inhibitor SB-201076, was proved to have a hypolipidemic effect (Pearce et al., 1998). Later, further cell experiments and animal models showed that SB-204990 had significant proliferation inhibition in lung cancer and prostate cancer (Hatzivassiliou et al., 2005). In 2017, Koerner et al. (2017) developed a series of ACLY inhibitors with chemical structures similar to that of anthraquinone emodin, which can induce apoptosis of non-small cell lung cancer cells by knocking down ACLY, thus achieving anti proliferation effect. In general, the ACLY inhibitors are still in the preclinical research, but there is no denying that their great effects in inhibiting cancer. Moreover, recent studies have indicated the crucial role of acetyl-CoA in cancer metabolic pathways, suggesting that targeting acetyl-CoA metabolic pathway mediated by ACLY is likely to become a new and efficient anticancer strategy. By analysing differences in acetyl-CoA metabolism between cancer cells and normal cells, new ways to fight cancer may be discovered.

### Cancer therapy mediated through other pathways

In fact, acetyl-CoA can profoundly affect cancer occurrence and progression through metabolic pathways, and the effect is not limited to that on ACLY. Research has shown that pyruvate kinase M2 (PKM2) reduces glucose consumption and citrate production in cells, promotes reductive glutamine metabolism to produce acetyl-CoA, and provides the basis for cancer cell proliferation by maintaining proton homeostasis (Liu et al., 2018). In acute myeloid leukemia (AML), AMP-activated protein kinase (AMPK) deficiency can cause an imbalance in acetyl-CoA homeostasis and reduce histone acetylation to inhibit AML development (Jiang et al., 2019). Similarly, two other studies have shown that inhibiting downstream acetyl-CoA carboxylase in the metabolic pathway can effectively prevent the initiation of fatty acid synthesis and increase the mitochondrial oxidation rate to inhibit the growth of non-small cell lung cancer (NSCLC) and glioblastoma (Svensson et al., 2016; Jones et al., 2017). In addition, the targeted inhibition of pyruvate dehydrogenase kinase (PDK) changes pyruvic acid metabolism from lactic acid production to acetyl-CoA production and the tricarboxylic acid cycle. This increases mitochondrial reactive oxygen species generation and induces cancer cell apoptosis (Bonnet et al., 2007). As one of the main metabolic precursors of acetyl-CoA, citrate also plays a considerable anticancer role. Through intraperitoneal administration, citrate has shown a significant inhibitory effect on gastric cancer (Wang et al., 2016). Similarly, acetate, another major precursor of acetyl-CoA, potentiates apoptosis and anti-proliferative effect of colorectal cancer cells combined with glycolytic inhibitor 3-bromopyruvate. Therefore, maintaining a specific acetate concentration may be a complementary strategy in the current treatment of colorectal cancer (Ferro et al., 2016; Tran et al., 2021). Moreover, previous studies have shown that glyceryl triacetate (GTA)-mediated acetate supplement is a new therapeutic method to inhibit the growth of glioblastoma, which can provide a safe and effective epigenetic therapy without affecting the proliferation of normal neural stem cells (Long et al., 2013; Tsen et al., 2014; Long et al., 2015). In addition, ACSS2 plays an important role in synthesis of acetyl-CoA and inhibition of ACSS2 produces anti-cancer effects. A recent study showed that ACSS2 contributes to myeloma progression in obese patients and an ACSS2 inhibitor can reduce the growth of myeloma *in vitro* and in diet induced obese mouse models (Li et al., 2021). In breast cancer cells with long-term estrogen deprivation, the treatment of ACSS2 inhibitor leads to significant loss of viability and proliferation (Calhoun et al., 2022). Moreover, Gao et al. (2016) found that under hypoxia, ACSS2 in cancer cells consumes acetate to synthesize acetyl-CoA and then regulates the acetylation of histones H3 at lysine 9 (H3K9), H3K27 and H3K56 in the promoter region of adipogenesis-related genes. Then, it upregulates the transcription of fatty acid synthesis genes and promotes fatty acid production and cancer cell proliferation. In addition, experiments have shown that key enzymes in the acetyl-CoA metabolic pathway exert a profound impact on cancers (Table 1).

**TABLE 1:** Key enzymes in the acetyl-CoA metabolic pathway with a profound impact on cancers.

Enzyme	Cancer type	Effect	Methods references
ACC	Lung cancer, colorectal cancer, colon adenocarcinoma, etc. pancreatic cancer	Spiropentacylamide derivatives as ACC inhibitors may suppress tumor growth through increasing mitochondrial oxidation rates	Cell (Wei et al., 2018) culture
		By using an ACC inhibitor (BAY ACC002), the proliferation of pancreatic cancer cells is slowed *in vivo* and *in vitro*, and the growth of pancreatic cancer is suppressed	Cell culture, (Petrova et al., 2017) animal models
ACSS2	Breast cancer	An increased number of ACSS2 copies is associated with cancer progression and VY-3-135, an inhibitor of ACSS2, can inhibit tumor growth at a relatively low concentration	Cell culture, (Schug et al., 2015; Miller et al., 2021) animal models
	Glioblastoma	Phosphorylation of ACSS2 on serine 267 stabilizes ACSS2 protein levels and contributes to glioblastoma growth	Cell culture (Ciraku et al., 2022)
	Pancreatic cancer	ACSS2 knockout markedly inhibits cancer cells proliferation and the prolongation of survival in orthotopic mouse models	Cell culture, (Zhou et al., 2022) animal models
FASN	Breast cancer	Fasnall, a FASN inhibitor, has shown potential anticancer activity through the induction of apoptosis, especially when combined with carboplatin	Cell culture, (Alwarawrah et al., 2016) animal models
	NSCLC, ovarian cancer, and breast cancer	For multiple cancers, the disease control rate based on a single FASN inhibitor administered alone reached 42%, and in trials of a FASN inhibitor used in combination with paclitaxel, the rate reached 70%	Clinical (Falchook et al., 2021) studies
	Hepatocellular carcinoma	Stabilization of FASN through the ACAT1-GNPAT-FASN axis greatly contributes to the development of hepatocellular carcinoma	Cell culture, (Gu et al., 2020) animal models
GLUD1	Lung cancer, breast cancer, and leukemia	Targeting GLUD1 with the inhibitor r162 led to imbalanced redox and cancer cell proliferation	Cell culture, (Jin et al., 2015) animal models
	Glioblastoma	Under hypoglycemic conditions, GLUD1 upregulates glucose transporters and promotes glucose uptake and cancer formation	Cell culture, (Wang et al., 2019b) animal models and clinical studies
	Kidney renal clear cell carcinoma (KIRC)	GLUD1 is degraded following amino acid deprivation, thus inhibiting RP gene expression and retaining nutrition to maintain cancer cells growth	Cell culture, (Shao et al., 2021) animal models

Compared with the ACLY inhibitors, the researches of ACC inhibitors and ACSS2 inhibitor in cancers need to be increased. Soraphen A is a natural ACC inhibitor against the biotin carboxylase (BC) domain (Shen et al., 2004). In breast cancer dissemination and xenograft mice experiments, soraphen A abrogated the formation of lung metastasis whereas control cells disseminate to the lungs (Stoiber et al., 2018). In addition, soraphen A was proved to inhibit breast cancer stem cells population through ACC driven adipogenesis (Corominas-Faja et al., 2014). Recently, ND-654, a novel ACC inhibitor, has been found to inhibit hepatocarcinogenesis by interfering with liver *de novo* lipogenesis (Lally et al., 2019). On the contrary, there are few studies on the use of an ACSS2 inhibitor to block cancer growth. Recently, it was reported that a newly synthesized small molecule inhibitor, as a transition state mimic, effectively impairs the growth of breast cancer (Miller et al., 2021). Interestingly, another trial showed that rapamycin may also inhibit the growth of breast cancer through ACSS2 target (Liang et al., 2021). In addition, tetrazole and novel amide-substituted condensed pyridine derivatives have recently applied for cancer treatment as two new ACSS2 inhibitors (Sabnis, 2021a; Sabnis, 2021b). Unfortunately, most of the FASN inhibitors have good anti-cancer effects, but the side effects hinder thier advancement in clinical trials. Cerulenin is a small molecule inhibitor of FASN that showed anticancer effect on breast cancer and ovarian cancer in the early stage (Pizer et al., 1996a; Pizer et al., 1996b), but its off-target activities hindered its clinical development (Angeles and Hudkins, 2016). Another well-known drug used to treat obesity, orlistat, can shrink tumors in non-small cell lung cancer and in pancreatic cancer (Sokolowska et al., 2017; Ali et al., 2018), but its progress in clinical trials is hindered by a series of reasons such as high instability, poor gastrointestinal absorption, etc (Zhi et al., 1995). At present, only TVB-2640 is the first clinically tested selective and potent FASN inhibitor, and is being included in the clinical trial (ClinicalTrials.gov: NCT02223247) of paclitaxel combined cohorts in cancer treatment (Patel et al., 2015).

Therefore, the acetyl-CoA metabolic pathway seems to be a vital pathway in life activities and cancer treatment. By comparing and analysing these research results, we ask the following question: Can an anticancer treatment be more effective by altering multiple metabolic pathways to inhibit acetyl-CoA production? We think that the answer is yes and it is worth encouraging researchers to develop a greater number of more effective cancer treatments by considering strategies that combine multiple acetyl-CoA inhibition pathways.

## Roles of epigenetic changes mediated through acetyl-CoA pathways in cancer

Cancer formation involves complex physiological mechanisms, including changes in the microenvironment, metabolism, and genetic material. Recently, abnormal epigenetic changes have been found to play a profound role in cancer development. Epigenetic modifications do not change DNA sequences. However, genetic functions undergo heritable changes and eventually lead to phenotypic changes (Dupont et al., 2009). Epigenetic modifications mainly include histone modification, which includes methylation, acetylation, and phosphorylation, DNA methylation, chromatin remodeling and noncoding RNA regulation (Zhang et al., 2021). In this section, we discuss the role of histone acetylation mediated by acetyl-CoA in cancer formation and growth.

Histone acetylation is regulated by the opposite activities of HATs and histone deacetylases (HDACs), both of which are closely related to cancer prognosis and treatment. Histone acetylation is catalyzed by histone acetyltransferases. At least 5 different HAT families have been discovered. The overall structures and catalytic outcomes differ, but the general process involves the transfer of acetyl groups from acetyl-CoA to lysine residues (N-terminus) and the production of CoA (Yuan and Marmorstein, 2013). Acetylation reduces the positive charge of amino acid residues, inhibits the binding of histone tails to negatively charged DNA, and exposes the underlying DNA. Therefore, acetylation can be considered an active labelling method (Bannister and Kouzarides, 2011). HDACs are critical for the deacetylation process of histones, and they contain 4 categories and 18 gene families. Among these HDACs, those in categories I, II and IV are known as classic HDACs, which have been recently identified as anticancer drug targets (Witt et al., 2009). A multiomic prognostic analysis of glioma acetylation regulators showed that lysine acetylation may lead to the malignant progression of glioma, and the observed changes in several lysine acetylation regulators, including HDACs and HATs, showed prognostic value in gliomas (Tu et al., 2021). Specifically, studies have shown that the expression of a variety of specific HDACs in cancer cells is upregulated and associated with poor prognosis. New research suggests that HDAC6/SP1 axis activation is associated with poor clinical outcomes in patients with glioblastomas or low-grade gliomas (Yang et al., 2020b). A previous study showed that although HDAC3 expression varies greatly among individuals with lung cancer, it is significantly upregulated in cancer tissues compared with normal lung tissue (Bartling et al., 2005). The same pattern has been found in gynecological malignancies, as high levels of HDAC expression have been observed in ovarian and endometrial cancers. In endometrioid tissue subtypes, the expression of three class I HDACs has been shown to be an independent prognostic marker (Weichert et al., 2008). HDAC inhibitors have been widely used in cancer treatments. In the past, they have been mainly used to treat nonsolid cancers such as hematopoietic malignancies and mental diseases, but have shown poor curative effects in solid cancers. In recent years, HDAC inhibitors have been approved for the treatment of cutaneous T-cell lymphoma (CTCL) (Khan and La Thangue, 2008), and their efficacy on solid cancers has been examined. In phase I/II trials, the combination of azacytidine and HDAC inhibitors showed encouraging results in refractory metastatic NSCLC (Rodríguez-Paredes and Esteller, 2011). In another study, the combination of an HDAC inhibitor with carboplatin and paclitaxel achieved good efficacy in pretreated patients with advanced epithelial ovarian cancer, showing an overall response rate of 43% and good tolerance (Dizon et al., 2012). HDAC inhibitors induce synergistic effects with BETs, and this combination shows a good inhibitory effect on pancreatic cancer (He et al., 2020). Interestingly, although HDAC inhibitors in combination with other drugs have been gradually indicated to be effective in some solid cancers, the effect of HDAC inhibitors alone has been disappointing.

In contrast to HDAC inhibitors, HAT inhibitors are in the very early stage of development because they lack high selectivity and power as inhibitors. Previous studies have proven that HAT inhibitors show potential to treat cancer. For example, the panhat inhibitor pu139 showed a significant anticancer effect on neuroblastoma xenografts *in vivo* (Gajer et al., 2015). As inhibitors of kat6a and kat6b (HATs in the MYST family), wm-8014 and wm-1119 induced cell senescence *in vitro* and in zebrafish hepatocellular carcinoma models and prevented the progression of mouse lymphoma (Baell et al., 2018). Moreover, recent studies have reported that small-molecule inhibitors can specifically act on the catalytic activity of p300/CBP (two important HAT family members) and show high potency and selectivity (Lasko et al., 2017; Yang et al., 2020c). Although HAT treatments are still preliminary, HAT use in the prognosis of some specific cancers has been promising. For example, the expression of hmof, an H4K16-specific HAT, is significantly lower in primary breast cancer and medulloblastoma. In medulloblastoma, the downregulation of hmof protein expression has been associated with poor survival and may play a role as a prognostic marker (Pfister et al., 2008). Another study showed that the deletion of KAT7 can lead to a decrease in AML cell proliferation and an increase in apoptosis and differentiation, but unfortunately, a relationship between AML cell depletion and AML prognosis has not been proven (Au et al., 2021). Nevertheless, these results show that HAT, as one of the most important enzymes regulating histone acetylation, has not been sufficiently developed or utilized in cancer applications. In fact, epigenetic targeted therapy has shown viable therapeutic potential in cancer treatment and is not limited to the regulation of histone acetylation related enzymes, but also the development of new drugs for DNA methylation and histone methylation related enzymes (Cheng et al., 2019). In many clinical trials, epigenetic regulators developed for mutation or translocation proteins have achieved good efficacy in cancer (Mohammad et al., 2019). However, it is obvious that the treatment of cancer by regulating histone acetylation related enzymes has not been effectively developed. Therefore, we suggest focusing on the synergistic effects of HAT inhibitors, HDAC inhibitors and other anticancer drugs. That is, if cancer can be treated or the prognosis of cancer can be predicted through acetylation regulation, then more alternative pathways will be available for use in future treatments.

To date, relevant biomarkers are lacking for a large proportion of cancers. If we can find specific markers for the timely or early detection of cancer, we can certainly reduce mortality associated with a large proportion of cancers. Epigenetic changes show stability and are measurable in readily available bodily fluids. These characteristics make them likely to become the next generation of cancer markers. Existing studies report epigenetics-based diagnosis and prognostic markers in several common cancers that mostly involve noncoding RNA or DNA methylation. This means few acetylation markers are being used for cancer diagnosis or prognostic monitoring, and the existing acetylation markers predict cancer prognosis through histone H3. Here, we summarize the application of existing histone acetylation biomarkers in cancer (Table 2). In one previous study, a low acetylation level of H3K18ac was found to be a separate, potentially important prognostic marker that is inversely proportional to breast cancer grade (Elsheikh et al., 2009). H3K18ac has also been associated with poor prognosis in HCC. Low H3K18ac expression is an independent and valuable prognostic factor in HCC patients (Lee et al., 2016). Similarly, increased acetylation levels of H2BK120ac, H3.3K18ac and H4K77ac in HCC patients were significantly related to poorer survival and higher recurrence (Chai et al., 2021). Another study showed that the expression level of an acetylation-related long noncoding RNA, AC099850.3, was significantly elevated in NSCLC tissues and cells, and it may serve as a potential biomarker for the prognosis of NSCLC patients (Zhou et al., 2021). In a recent liver cancer study, gene manipulation through acyl-CoA thioesterase 12 (ACOT12) led to significant changes in H3 acetylation in different HCC cell lines, making it useful as a prognostic marker (Lu et al., 2019). In addition, the key enzymes in the acetylation pathway can actually be used as markers for monitoring prognosis and have a good performance. For example, ACSS2 contributes to obesity induced myeloma and patients with high levels of ACSS2 expression in myeloma cells have shorter overall survival than patients with low expression. The expression of ACSS2 in myeloma cells of patients with malignant subtypes was significantly higher than that of patients with subtypes of good prognosis (Li et al., 2021).

**TABLE 2:** Summary and supplement on changes of histone acetylation biomarkers of cancers.

Histone acetylation biomarkers	Cancer types	Targeted therapy	References
H2BK5	Neuroblastoma	Benzamide-containing HDAC inhibitor	Lauffer et al. (2013)
H4K5	Head and neck squamous cell carcinoma	Vorinostat (HDAC inhibitor)	Milan et al. (2022)
H4K12	Colorectal cancer	Suberoylanilide hydroxamic acid (HDAC inhibitor)	Alzoubi et al. (2016)
H4K16	Tumorigenic human prostate epithelial cells; Colorectal cancer	Lunasin (histone acetylation inhibitor); Suberoylanilide hydroxamic acid (HDAC inhibitor)	(Galvez et al., 2011; Alzoubi et al., 2016)
H3K4	Endometrial carcinoma	RGFP-966 (HDAC3 inhibitor)	Chen et al. (2022a)
H3K9	Prostate cancer	Panobinostat (HDAC inhibitor)	Chen et al. (2022b)
H3K18	Hepatocellular Carcinoma; Breast cancer; Kidney cancer and clear cell renal carcinoma	B029-2 (acetyltransferase inhibitor)	(Elsheikh et al., 2009; Lee et al., 2016; Cocco et al., 2018; Cai et al., 2021)
H3K27	Gliomas	Panobinostat (HDAC inhibitor)	Krug et al. (2019)
H3K36/H3K79	Head and neck squamous cell carcinoma	Vorinostat (HDAC inhibitor)	Milan et al. (2022)

**FIGURE 4:**
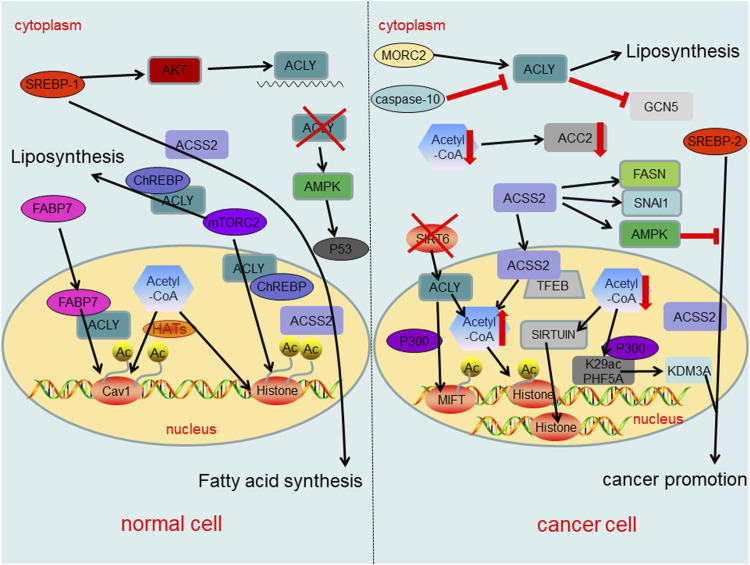
Summary of regulatory roles of acetyl-CoA metabolism and key enzymes in acetyl-CoA metabolic pathway both in normal cells and cancer cells.

In fact, the changes of acetyl-CoA metabolism and its related enzymes have different regulatory effects in cancer cells and normal cells, thereby affecting epigenetics as well as cancer formation (Figure 4). Dramatic reduction of acetyl-CoA in cancer cells results in sirtuin-mediated histone deacetylation and downregulation of ACC2 (Corbet et al., 2016). Similarly, PHF5A, a component of small nuclear ribonucleoprotein, can be acetylated at lysine 29 dependent on p300 and promotes KDM3A protein expression, leading to poor prognosis of cancer (Wang et al., 2019c). In normal cells, ACLY expression is mainly regulated by the transcription factor SREBP-1 (sterol regulatory element binding protein-1) which up-regulates ACLY at mRNA level *via* AKT signaling (Sato et al., 2000; Bauer et al., 2005). Another intracellular fatty acid chaperon that interacts with ACLY is fatty acid binding protein 7 (FABP7), which leads to histone acetylation of several gene promoters including caveolin-1 (Kagawa et al., 2020). Evidences also suggest that mammalian target of rapamycin complex 2 (mTORC2) acts through ACLY to increase carbohydrate response element binding protein (ChREBP) activity to synthetic lipid and histone acetylation. Additionally, mTORC2 also promotes acetyl-CoA synthesis from acetate through ACSS2 (Fernandez et al., 2019; Martinez Calejman et al., 2020). In cancer cells the inactivation of SIRT6 leads to the accumulation of nuclear ACLY protein, increases the pool of nuclear acetyl-CoA, and then drives the histone acetylation and cancer invasion (Zheng et al., 2021a). In addition, ACLY enhanced the activity of acetyltransferase P300, increasing the histone acetylation at MITF locus to promote MITF-PGC1α axis transcription (Guo et al., 2020). In the cytosol of cancer cells, ACLY activation has been promoted by Microrchidia (MORC) family CW-type zinc finger 2 (MORC2) to regulate lipid homeostasis (Sánchez-Solana et al., 2014). Intracytoplasmic caspase-10 mediated ACLY cleavage leads to the decrease of intracellular lipid level, and inhibits (general control nonderepressible 5) GCN5 and its mediated histone H3 and H4 acetylation, resulting in the down-regulation of proliferation and transfer genes (Kumari et al., 2019). Meaningfully, the activation of AMP-activated protein kinase (AMPK) under ACLY silencing conditions may lead to cancer suppressor p53 activation, ultimately leading to DNA damage-induced cell death both in primary human cells and cancer cells (Lee et al., 2015). Also, ACLY might represent a promising target in which ACLY inhibitor BMS-303141 could induce ER stress and activate p-eIF2α/ATF4/CHOP axis to promote apoptosis of HCC cells (Zheng et al., 2021b). In normal cells, the expression of ACSS2 is regulated by sterol regulatory element binding protein 1 (SREBP-1), which can regulate fatty acid synthesis (Xu et al., 2018). On the other hand, SREBP-2 targets ACSS2 to provide a growth advantage to cancer cells in the condition of the tumor microenvironment (Kondo et al., 2017). Under nutritional stress conditions, acetate increased the expressions of SNAI1 and ACSS2. Also, ACSS2 overexpression increased SNAI1 level, H3K27ac and FASN expression (Gao et al., 2016; Yao et al., 2020). Similarly, ACSS2 induces apoptosis of cancer cells by activating AMPK (Mi et al., 2019). Another study shows that glucose deprivation results in the binding of transcription factor EB (TFEB) and ACSS2 to the promoter regions of CTSA, GBA, GUSB, and LAMP1 and utilizes the acetate to produce acetyl-CoA locally for histone acetylation (Li et al., 2017).

These studies suggest that future research should be directed towards the cancer based on epigenetic biomarker because it is very different between cancer cells and normal cells and its analyses and treatment schemes may be powerful weapons against cancer. Research on acetylation regulation in cancer is currently focused on the treatment of cancer through HATs and HDACs, resulting in the rare use of acetylated biomarkers in cancer diagnostics and prognostics. However, as stable and easily available markers, epigenetic cancer biomarkers may be particularly useful in cancer diagnostics and prognostics. In summary, epigenetic modifications comprise a very promising field worthy of exploration by more scholars.

## Conclusion

Since they have been properly defined, cancers have been identified as a main cause of death worldwide, and after many years of exploration into its causes and treatments, the fatality rate is still very high. Currently, increasing attention is being focused on the diagnosis and treatment of cancer, and increasingly, scientific and technological strategies are being applied to the treatment of cancer. However, advancements in precise surgical treatments and local radiotherapies have been difficult. In contrast, recent progress in molecular biology has led to an increasing number of metabolic pathways in cancers being explored for diagnosis and treatment, and acetyl-CoA metabolic pathways are among these pathways receiving increased attention. In this review, we first discussed the normal pathways of acetyl-CoA metabolism and their abnormal regulation in cancers, and we explained that acetyl-CoA metabolism differences in cancer and normal cells may provide clues for cancer diagnosis. Second, through the analysis of many different methods for blocking the metabolism of acetyl-CoA, we think that treatments directed at a combination of these pathways may lead to effective cancer therapies. Finally, we discussed the effect of acetylation on cancer cells and found that most studies currently focus on the treatment of HATs and HDACs. However, they ignore the possibility of using epigenetic biomarkers as more effective sources of cancer diagnostics and prognostics. In general, there is still a long way to go in overcoming cancer, and exploring new and more effective ways to treat it remains a top priority. We hope that some ideas presented in this review advance research on cancer diagnosis and treatment-related aspects in the future.
